# Detecting Air Pollutant Molecules Using Tube-Shaped Single Electron Transistor

**DOI:** 10.3390/molecules26237098

**Published:** 2021-11-24

**Authors:** Zhongkai Huang, Xiangyang Peng, Cheng Peng, Jin Huang, Maolin Bo, Chuang Yao, Jibiao Li

**Affiliations:** 1 School of Chemistry and Chemical Engineering, Chongqing Key Laboratory of Soft-Matter Material Chemistry and Function Manufacturing, Southwest University, Chongqing 400715, China; zhongkaihuang@yznu.edu.cn; 2 Key Laboratory of Inorganic Special Functional Materials of Chongqing, Yangtze Normal University, Chongqing 408100, China; 3 Hunan Key Laboratory of Micro-Nano Energy Materials and Devices, Xiangtan University, Xiangtan 411105, China; 4 Key Laboratory of Extraordinary Bond Engineering and Advanced Materials Technology of Chongqing, Yangtze Normal University, Chongqing 408100, China; bmlwd@yznu.edu.cn (M.B.); yaochuang@yznu.cn (C.Y.); jibiaoli@yznu.edu.cn (J.L.)

**Keywords:** tube-shaped single electron transistors, incoherent transport, air pollutant molecule, first-principle calculations, identification and quantification

## Abstract

An air pollution detector is proposed based on a tube-shaped single-electron transistor (SET) sensor. By monitoring the flow control component of the detector, each air pollutant molecule can be placed at the center of a SET nanopore and is treated as an island of the SET device in the same framework. Electron transport in the SET was incoherent, and the performances of the SET were sensitive at the single molecule level. Employing first-principles calculations, electronic features of an air pollutant molecule within a tube-shaped SET environment were found to be independent of the molecule rotational orientations with respect to axis of symmetry, unlike the electronic features in a conventional SET environment. Charge stability diagrams of the island molecules were demonstrated to be distinct for each molecule, and thus they can serve as electronic fingerprints for detection. Using the same setup, quantification of the air pollutant can be realized at room temperature as well. The results presented herein may help provide guidance for the identification and quantification of various types of air pollutants at the molecular level by treating the molecule as the island of the SET component in the proposed detector.

## 1. Introduction

Air pollution occurs when harmful or excessive quantities of substances are introduced into Earth’s atmosphere, leading to public health and environmental problems [1]. On the public health side, the toxic effects of air pollution have been individually identified in various organs of the body, leading to eighteen outpatient diseases, including cancer [2]. In addition to the documented physical effects of air pollution on humans, there are negative effects of air pollution on subjective well-being [3]. On the environmental side, air pollution can damage ecosystem functions and structures and result in global warming, acid rain, and deterioration of the ozone (O${}_{3}$) layer [4]. The impact of air pollution on materials is also notorious, as chemical reactions between the polluted air and material matrices coating buildings or within structures may result in large maintenance costs [5].

Air pollutants are the source of air pollution, and they can be classified as “criteria pollutants” and “hazardous air pollutants” [6]. Criteria pollutants are used to determine if one region meets air quality standards. The most common criteria pollutants include particulate mater (PM), carbon monoxide (CO), nitrogen dioxide (NO${}_{2}$), tropospheric ozone (O${}_{3}$), and sulfur dioxide (SO${}_{2}$) [7]. Hazardous air pollutants, known as “air toxics”, are chemical species that may cause cancer and other chronic human health risks. Frequently encountered hazardous air pollutants include benzene (C${}_{6}$H${}_{6}$), formaldehyde (CH${}_{2}$O), toluene (C${}_{7}$H${}_{8}$), xylene (C${}_{8}$H${}_{10}$), and benzo (a) pyrene (as a marker for polycyclic aromatic hydrocarbons) [8].

Various approaches have been developed to identify and quantify air pollutants in the atmosphere and determine the air quality. Direct and indirect measurements are used. Direct measurements include the sizes and concentrations of particles caught on filters, the concentrations of gases collected in traps, the pH values of liquid droplets, the temperature, and the humidity. Other measurements are indirect. For example, light scattering methods are used to determine the number of aerosol particles in the air [9].

However, as air pollutants have various types, individual detection methods must be tailored to specific features of the measured species. For instance, a primary method to detect carbon monoxide is based on nondispersive infrared photometry. The current method to identify sulfur dioxide employs ultraviolet fluorescence. Methane and other non-methane volatile organic compounds are measured using gas chromatography with a calibrated flame ionization detector [9]. Therefore, more technologically advanced methods are necessary to unify the measurements of various air pollutant species.

Given the wide application of semiconductors in gas sensing, the use of semiconductor devices to measure air pollution is promising. In recent decades, low-dimensional nanostructures have received considerable attention for gas sensing, as the resistance changes drastically due to the absorption of foreign molecules on materials with high surface-to-volume ratios [10]. High gas-sensing abilities have been found in low-dimensional nanostructures, such as carbon nanotubes [11], ZnO nanobelts [12], silicon [13], In${}_{2}$O${}_{3}$[14], and SnO${}_{2}$[15] nanowire-based sensors, and two-dimensional materials, such as graphene [16] and MoS${}_{2}$ [17]. However, those sensors are mostly based on coherent electronic transport [18]. Inevitably, electric heat will be produced during the operation of these sensors, and their large power costs will affect the high sensitivities of the sensors.

Based on sequential transport, single-electron transistors (SETs) can avoid the aforementioned problems induced by the coherent transport [19]. A SET consists of source, drain, and gate electrodes and an island [20]. There are tunnel barriers between the source/drain electrode and the island, and electronic conduction takes place via sequential quantum tunneling through the barriers. The gate electrode is designed such that the electrostatic potential of the island is in a Coulomb blockade state. Based on the scanning values of the gate voltage ($V_{g}$) and source–drain bias ($V_{b}$), a phase diagram called the charge stability diagram can be obtained to unveil the conduction behaviors of the electrons in an SET [21,22]. According to the underlying physics of the physical quantities observed, potential applications of SETs have been found in logic operations [23,24], quantum computation [25], and sensing [26,27,28]. Gas molecules have been proposed to be the island of the SET for sensing. Ray et al. studied a series of molecules using SET with conventional structures and investigated the effects of gates tuning on the performance of SET [29,30,31,32,33,34]. Recent studies have shown that the types of island molecules can be identified by unique electronic signals from corresponding SET devices [34]. However, previous SET schemes were not designed for the purpose of air pollutant detection and cannot adequately meet the requirements for the identification and quantification of air pollutants at a molecular level.

In the present work, an air pollutant measurement system was designed based on a tube-shaped SET sensor, which was the key component of the detector. Monitored by the flow control component, an air pollutant molecule could flow to the center of the SET nanopore and act as an SET island. The electronic properties of island molecules under the SET environment were investigated using first-principles-based density functional theory (DFT) [35]. In detail, we examined the performances of SET configurations by calculating the physical quantities of interest, including the total energies as functions of the gate voltage, energy density, molecular energy spectra, and the charge stability diagrams.

The remainder of the paper is structured as follows. In Section 2, we present the proposed measurement system and the calculation method. In Section 3.1, the influence of conventional and tube-shaped SET environments is studied for a certain molecule with various orientations. In Section 3.2, the electronic features, such as the charge stability diagrams, are characterized for the identification of individual air pollutant molecules. In Section 3.3, the quantification of the air pollutants is examined using the proposed device. Conclusions are drawn in Section 4.

## 2. Materials and Methods

### 2.1. System Description

**Figure 1:**
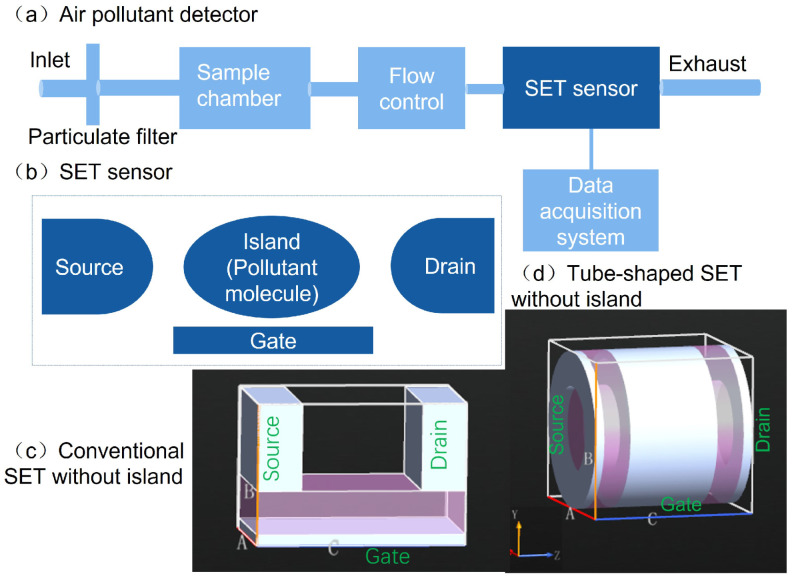
Schematic of an air pollutant detector based on a single-electron transistor (SET) sensor.

The proposed air pollution detector is depicted in Figure 1a. The setup consists of a particulate filter to clean particulate matter from the measured air, an air sample chamber to sample the ambient air, a flow control subsystem to move the sampled air into an SET sensor, an SET sensor with a suitable configuration, and a data acquisition subsystem for analyzing signals obtained from the SET sensor. In real application, the flow control subsystem would drive the molecules to small tubes whose sizes allow only single molecules to enter, and the molecule would then be precisely pulled through the pore in the SET sensor by a vertical electric field. The time for identifying a molecule included the time for pulling of the molecule into required positions in the sensor, time for completing an individual measurement, and the time for analyzing the measured data. During the measurement process, the molecule is treated as a static island of the SET device. Using the proposed setup, our objective is to guide the experimental development of the air pollutant detection using theory. Though it is not explicitly described here, it should be pointed out that other conditioning components are needed in experiments to minimize the measurement interference induced by the unsuitable flow control operations and to maximize the measurement processing ability.

As shown in Figure 1b, the geometry of the SET sensor included metallic source, drain, and gate electrodes and an island. If the coupling between the island and source/drain gates is strong, the coherent lifetime of the charge carrier is much longer than the propagating time on the island, and coherent transport thus dominates. If the island is weakly coupled to the source/drain gates, the charge carrier transfers from the source to the island and loses all information about its original quantum state due to sufficiently long staying time on the island. Together with a subsequent tunneling process into the drain electrode, the whole process is referred to as the sequential transport. As an example, Figure 1c,d plots conventional and tube-shaped SET structures without islands, respectively. The transport mechanism in the SET was sequential tunneling instead of coherent tunneling. Because of the weak coupling between the island and the source and drain electrodes, the electron moves through the SET and loses the information about its initial quantum state. In this transport step, the electron propagates independently from the drain electrode to the island and from the island to the source electrode. The gate potential of the gate electrode can tune the electron affinity levels and allows for opening and closing of the electron transport. Explicit principles for the SET operations are described in Appendix A.

**Figure 2:**
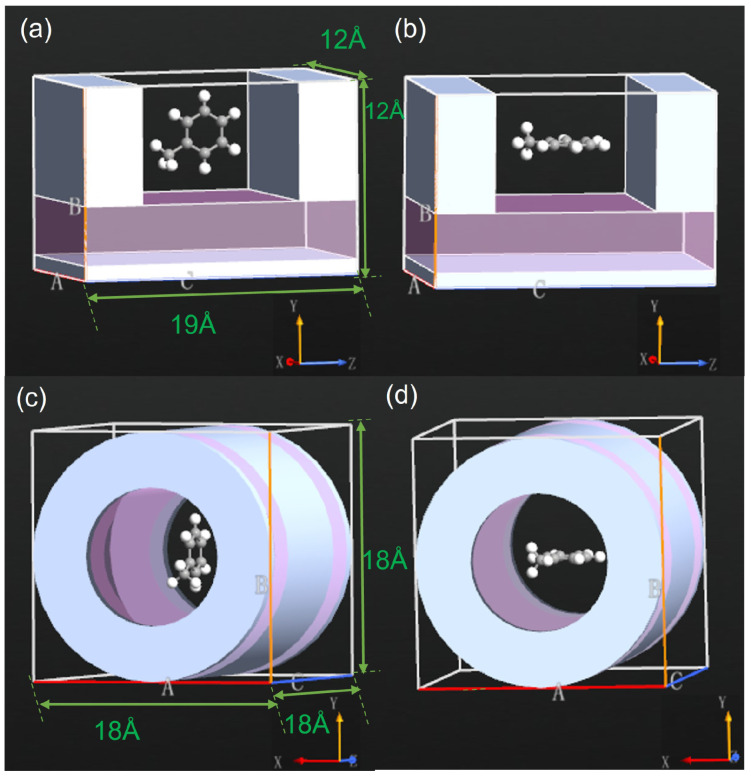
SET configurations with C${}_{7}$H${}_{8}$ as the SET island. The molecule was perpendicular to the in the left column (**a**,**c**) and parallel to *xz*-plane in the right column (**b**,**d**). The molecular center was aligned with the nanopore center.

Two types of SET structures are illustrated in Figure 2. A conventional structure is represented in the upper panels of Figure 2. The source and drain electrodes were at the two ends of the SET. Below these two electrodes was a dielectric layer with a dielectric constant of $10\varepsilon_{0}$ and a thickness of 4 Å. Beneath this layer, a gate electrode covered the entire area with a thickness of 1 Å. The three electrodes were metallic and used the work function of gold with a value of W=5.28 eV. To guide the experimental fabrication, these electrodes were considered to be made of other metals. The thickness along the *x*-axis was 12 Å for both the source and drain electrodes. The two electrodes had a horizontal separation of 11 Å along the *z*-axis. Along the *y*-axis, the two electrodes had the same height of 8 Å. The nanopore can be seen as a pore cross section with an area of 8 Å × 11 Å. The thickness of the whole SET structure was 12 Å along the *x*-axis. The size was comparable to those of earlier reported graphene- [36] and silicon-based [37] nanopores.

A tube-shaped SET structure is proposed in the current work, as shown in lower panels of Figure 2. This device was significantly different from the conventional one. The source and drain electrodes had the same metallic tube sections with inner radii of 5 Å, thicknesses of 4 Å, and lengths of 1 Å along the *z*-axis. The central part of the SET is the gate electrode with a length of 10 Å along the *z*-axis. The gate and source/drain electrodes were separated by a dielectric tube with a length of 3 Å at each end. The two dielectric tubes had the same inner radius and thickness as those of the source/drain electrodes. The tubular gate electrode had a thickness of 1 Å. The usage of 1 Å has been widely used, though it is even less than the radius of an atomic radius because it is sufficient to model the metal gate effect [33,34]. A dielectric layer with a thickness of 3 Å was placed underneath the gate and surrounded the nanopore of this SET structure. Compared to conventional SET devices, the newly proposed tube-shaped setup possessed several peculiar characteristics: (a) the source, drain, and gate electrodes were all tube-shaped, allowing a molecule to tunnel through the device before and after the measurements; (b) the gate electrode surrounded the entire channel and could provide stronger control over the electrostatics of the island compared to the conventional SET; and (c) the rotational angle along the the *z*-axis was flexible due to rotational symmetry.

A pollutant molecule was placed at the center of the nanopore as the SET island with various orientations to examine the capabilities of each SET structure. As a proof of principle, the C${}_{7}$H${}_{8}$ molecule was adopted in this section. The molecules in Figure 2a,c as well as Figure 2b,d were perpendicular to and parallel to the *xz*-plane, respectively. In all cases, the center of the molecule was placed at the center of the nanopore. In the experiments, the molecule entered the nanopore with various orientations relative to the gate. The SET responded correspondingly to the orientations, and the responses were examined. Influences of the orientation on the performance of the SET were revealed by investigating various physical properties of interest, such as the charge stability diagram.

### 2.2. Computational Procedure

Within the SET environment, the electronic properties of the air pollutant molecule were estimated using DFT calculations. The SET simulations not only used the pseudo-potential, but also introduced compensation charges at each atomic site to screen the electrostatic interactions. The method was implemented within the QuantumATK package [38], which performs calculations based on DFT and nonequilibrium Green’s function (NEGF) formalism. The DFT-NEGF-based methodology was developed [39,40] and introduced for non-equilibrium systems initially [41], and it was later expanded to the standard equilibrium case [42]. Stokbro completed this approach in the NEGF-DFT framework of Quantum Wise [35]. The self-consistent calculations employ the generalized gradient approximation of the Perdew–Burke–Ernzerhof exchange-correlation functional [43]. Metallic electrodes were used to fix the potential at a specified voltage on each electrode. To elucidate the absence of the perpendicular components of the electric fields from the metallic surfaces, Neumann boundary conditions were applied when solving the Poisson equation. This method has been successfully applied to estimate the charging energies of a variety of molecules within SETs [29,30,31,32,33,34,44,45].

## 3. Results and Discussion

### 3.1. Effects of SET Structure

**Figure 3:**
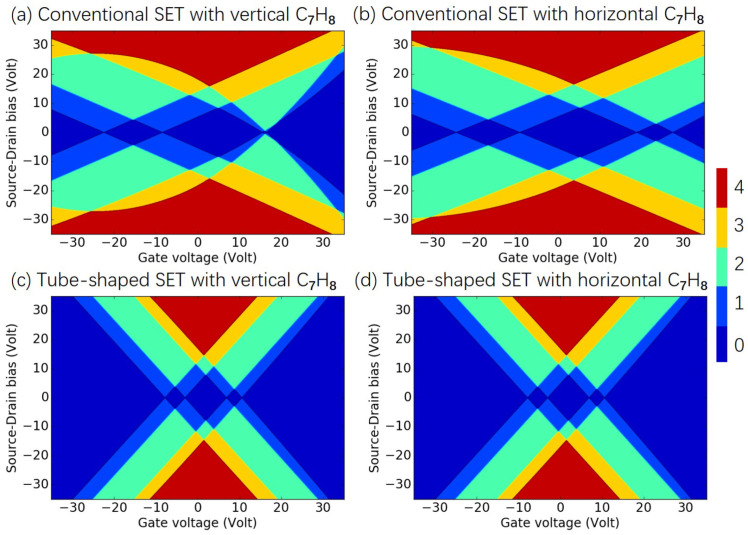
(**a**–**d**) Charge stability diagrams of various SET configurations for C${}_{7}$H${}_{8}$. For certain gate voltage and source–drain biases, the number of charge states within the bias window is indicated by color: red (4), yellow (3), green (2), blue (1), and dark blue (0).

As presented in Figure 3, the charge stability diagram illustrates the electrostatics and the nature of conduction within a SET in detail. If the island and source/drain electrodes are weakly coupled, conduction takes place via the way of sequential tunneling, where an electron goes from the source to the island and finally transfers to the drain so as to complete the conduction path. The island–source coupling and the island–drain coupling strengths determine the tunneling rate. In the case of an SET with a large source–drain separation, the incoming state of the electron is roughly uncorrelated with the outgoing state. Scanning a line along the gate voltage $V_{g}$ at a fixed source–drain bias $V_{d}$ would lead to a series of periodic peaks, which would indicate the addition of an electron to or removal of an electron from the island. If the scanning process in a symmetric range of $V_{d}$ was repeated, diamond-shaped regions would be found on the $V_{g}-V_{d}$ plane, which is known as the charge stability diagram. No conduction occurred within each of the diamond regions, and the charge population change by 1 between neighboring diamonds. Details of the mathematical descriptions of the charge stability diagram are interpreted in Appendix A. Therefore, the SET configurations with molecules of various orientations can provide charge stability diagrams with certain features. Figure 3a–d shows the charge stability diagrams of the configurations shown in Figure 2a–d, respectively. Figure 3a,b shows quite different features, indicating that the molecule orientation in a conventional SET can strongly affect the SET performance. In contrast, Figure 3c,d presents the same figure patterns, implying that the molecule orientation in the tube-shaped SET had a negligible influence on its charge stability diagram.

**Figure 4:**
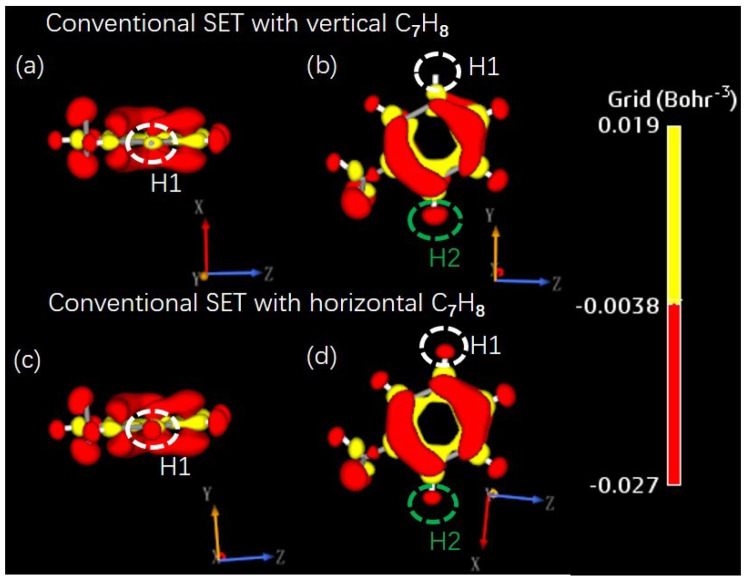
(**a**–**d**) Electron density of an additional net charge of +1 distributed on C${}_{7}$H${}_{8}$ under a conventional SET environment. The upper and lower panels represent the cases with vertical and horizontal C${}_{7}$H${}_{8}$ molecules, respectively. The charge clouds are illustrated from different viewpoints in the left and right columns.

Next, the electronic structures of the molecule were calculated to clarify the SET environmental effect on and the charging process of the island molecule. In the real space, the electron density of an isolated molecule can be altered by the SET electrostatic potential and the additional charge. Under the conventional SET environment, Figure 4 shows the distribution of electron density of an additional net charge of +1 on C${}_{7}$H${}_{8}$, i.e., the differences between electron densities of C${}_{7}$H${}_{8}$ with charge states of q=+1 and q=0. The major differences between the electron densities of molecules oriented vertically and horizontally are evident in the figure. For example, the electron density on atom H1 in the vertically positioned C${}_{7}$H${}_{8}$ was lower than that in the horizontal C${}_{7}$H${}_{8}$, as shown by the red regions inside the white circles in the upper and lower panels. In contrast, a greater electron density was found on atom H2 (inside the green circles) in the vertical C${}_{7}$H${}_{8}$ than in the horizontal C${}_{7}$H${}_{8}$. Under tube-shaped SET environment, the distributions of the additional net charge were the same for the vertical and horizontal C${}_{7}$H${}_{8}$, as shown in Figure A6 of Appendix D. This agrees with that fact that the charge stability diagram in the conventional SET relies on the orientation of the island molecule, while that in the tube-shaped SET was independent of the molecule orientation along the *z*-axis.

**Figure 5:**
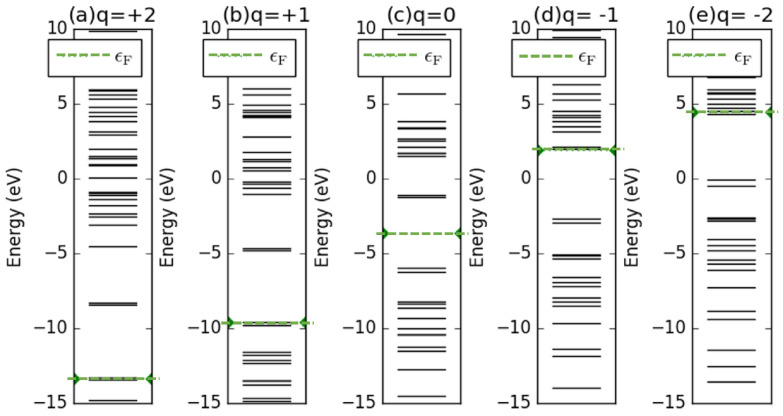
(**a**–**e**) Molecular energy spectra of vertically positioned C${}_{7}$H${}_{8}$ with various charge states under the tube-shaped SET environment. The fermion energy level is marked in each subplot with green dots.

In the energy space, we considered the vertically positioned C${}_{7}$H${}_{8}$ in the tube-shaped SET as an example to study the energy levels in various charging states, as illustrated in Figure 5. The molecular energy spectrum for the island molecule retained its structure during the operation of the SET device. However, the energy levels shifted by certain amounts to let the net charges enter or leave the island molecule. The energy levels entirely shifted upward when the island molecule was negatively charged, while they shifted downward as the electron moved away from the molecule. For example, if one electron was added to the island molecule, the originally lowest unoccupied molecular orbital (LUMO) in the neutral state shown in Figure 5c will become occupied by one electron, becoming the highest occupied molecular orbital (HOMO) in the charge state of q=−1, as presented in Figure 5d. Figure A7 in Appendix D presents the energy levels of the horizontally placed C${}_{7}$H${}_{8}$ in the tube-shaped SET, and the spectra were the same as those of the case discussed here. In addition, Figure A8 and Figure A9 in Appendix D show the energy levels of the vertical and horizontal C${}_{7}$H${}_{8}$ in a conventional SET, and they have same spectral structure as those of the cases in the tube-shaped SET. However, slight energy shifting occurred due to the electrostatic effect from the SET environment.

**Figure A3:**
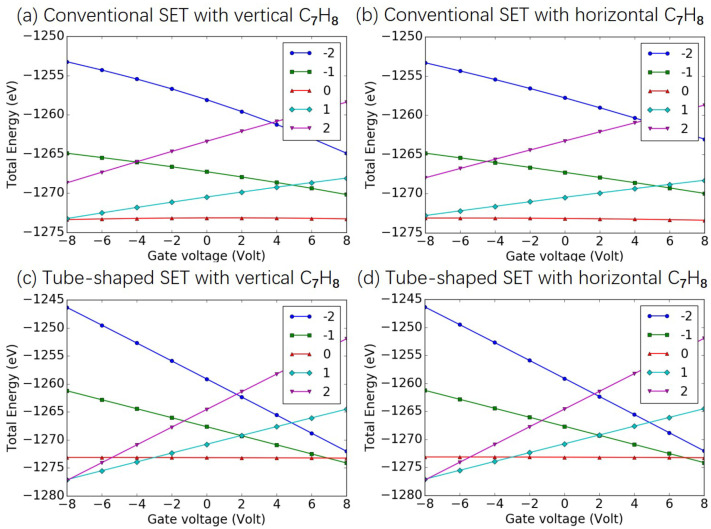
Total energy as a function of the gate voltage for C${}_{7}$H${}_{8}$ in the (**a**) conventional SET with the island molecule vertically oriented, (**b**) conventional SET with the island molecule horizontally oriented, (**c**) tube-shaped SET with the island molecule vertically oriented, and (**d**) tube-shaped SET with the island molecule horizontally oriented. Different curves correspond to charge states −2, −1, 0, +1, +2.

The dependence of the total energy on the gate potential was then studied to further understand the underlying electronic properties of the two types of SET structures. Based on the DFT calculations, the total energies of the island molecule within the SET environment were obtained for various charge states, as shown in Figure A3 in Appendix C. Using the calculated total energy, we fit a quadratic function as follows [32,35]:

(1) $$E\left(q,V_{g}\right)=E_{0}+qW+\alpha qV_{g}+\beta {\left(eV_{g}\right)}^{2},$$

in which $E_{0}$ denotes the zeroth-order term, which corresponds to a constant energy. The second term qW is the reservoir energy, where *q* represents the charge of the island molecule, *W* denotes the work function of the electrode, and a value of W=5.28 eV was used to model a gold electrode. The third term in Equation (1), which represents the direct coupling between the island molecule and the gate electrode, is proportional to the charge *q* on the molecule and is linear with respect to the gate voltage $V_{g}$. The linear coupling strength, α, depends on the relative position/orientation between the island and the gate for a certain type of molecule. The fourth term is independent of the charge state and has a quadratic dependence on the gate voltage $V_{g}$. The quadratic coupling strength, β, estimates the contribution of electrical polarization under the influence of an electric field. The values of the coupling strengths were estimated by making a least squares fit to the total energies from the DFT calculations, and those of the configurations in Figure 2 are listed in Table 1.

For conventional SET configurations shown in Figure 2a,b, the coupling strengths strongly depended on the orientation of the island molecule. As shown in Table 1, the vertically placed molecule caused stronger quadratic gate–island coupling than the horizontally oriented molecule. The strong dependence of the gate–island coupling on the orientation of the molecule in the SET suggested that the conventional SET is not suitable for the detection of pollutant molecules, as the molecules would arrive the nanopore with various orientations. As shown in the last two lines of Table 1, for the configurations shown in Figure 2c,d, the total energy was almost linearly dependent on $V_{g}$, indicating the minimal polarization contribution in the tube-shaped geometry. This minimum is possible because of the large coverage of the gate electrode on the island area from all different sides. The gate–island coupling strengths are identical for the configurations shown in Figure 2c,d. Thus, we concluded that the rotational symmetry of the tube-shaped device provided greater convenience for identifying the types of various molecules than the conventional SET. In the remainder of the manuscript, the tube-shaped SET will be employed to detect the pollutant molecules.

### 3.2. Identification of Air Pollutants

In this work, each air pollutant molecule served as the SET island and determined the performance of the SET. A slew of gas-phase air pollution molecules, including criteria pollutants and hazardous air pollutant molecules, were studied for the purpose of detection.

**Figure 6:**
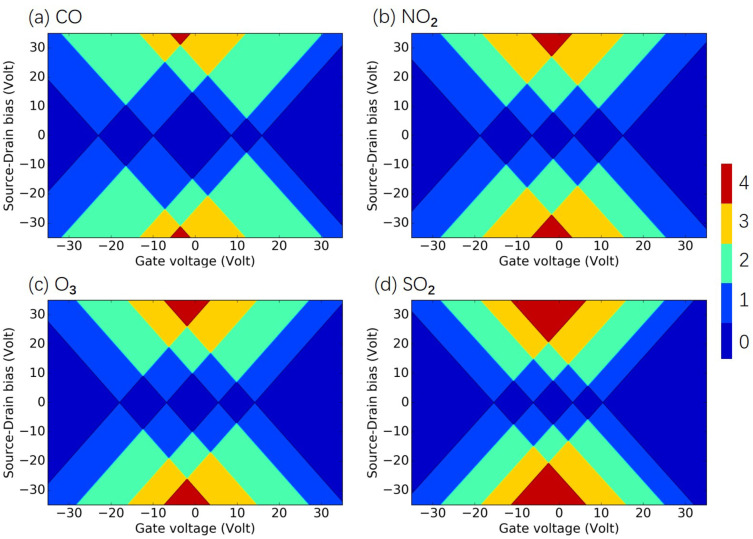
Charge stability diagrams of criteria pollutant molecules of (**a**) CO, (**b**) NO${}_{2}$, (**c**) O${}_{3}$, and (**d**) SO${}_{2}$. For certain gate voltage and source–drain bias, the number of charge states within the bias window is indicated by the color: red (4), yellow (3), green (2), blue (1), and dark blue (0).

**Figure 7:**
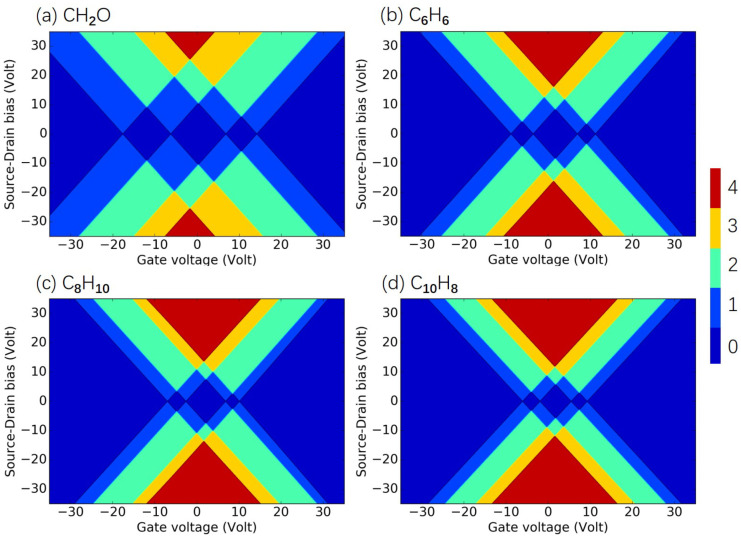
Charge stability diagrams of hazardous pollutant molecules of (**a**) CH${}_{2}$O, (**b**) C${}_{6}$H${}_{6}$, (**c**) C${}_{8}$H${}_{10}$, and (**d**) C${}_{10}$H${}_{8}$. For certain gate voltage and source–drain bias, the number of charge states within the bias window is indicated by the color: red (4), yellow (3), green (2), blue (1), and dark blue (0).

**Figure A4:**
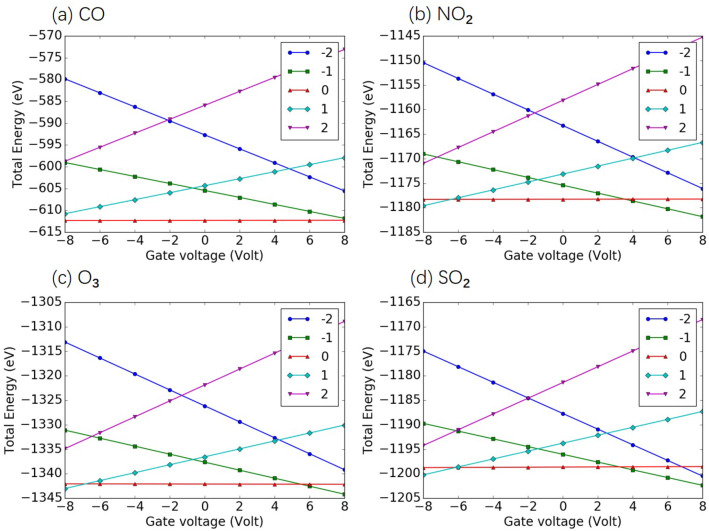
Total energy as a function of the gate voltage for criteria pollutant molecules (**a**) CO, (**b**) NO${}_{2}$, (**c**) O${}_{3}$, and (**d**) SO${}_{2}$ in the tube-shaped SET environment. Different curves correspond to charge states −2, −1, 0, +1, +2.

**Figure A5:**
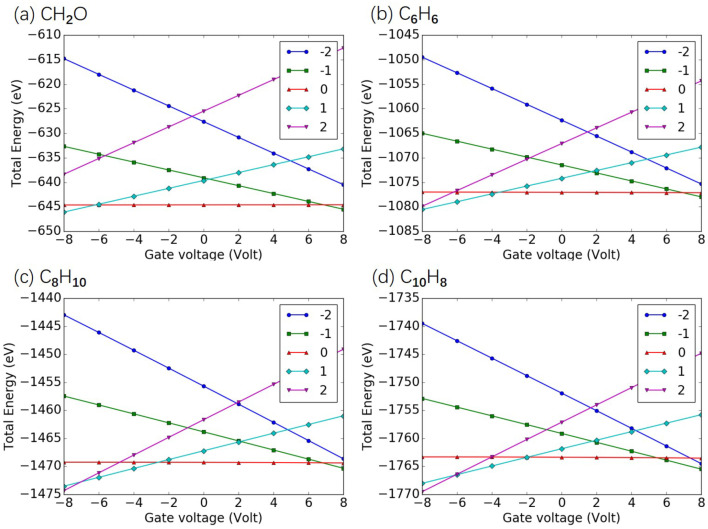
Total energy as a function of the gate voltage for criteria pollutant molecules (**a**) CH${}_{2}$O, (**b**) C${}_{6}$H${}_{6}$, (**c**) C${}_{8}$H${}_{10}$, and (**d**) C${}_{10}$H${}_{8}$ in the tube-shaped SET environment. Different curves correspond to charge states −2, −1, 0, +1, +2.

**Figure A6:**
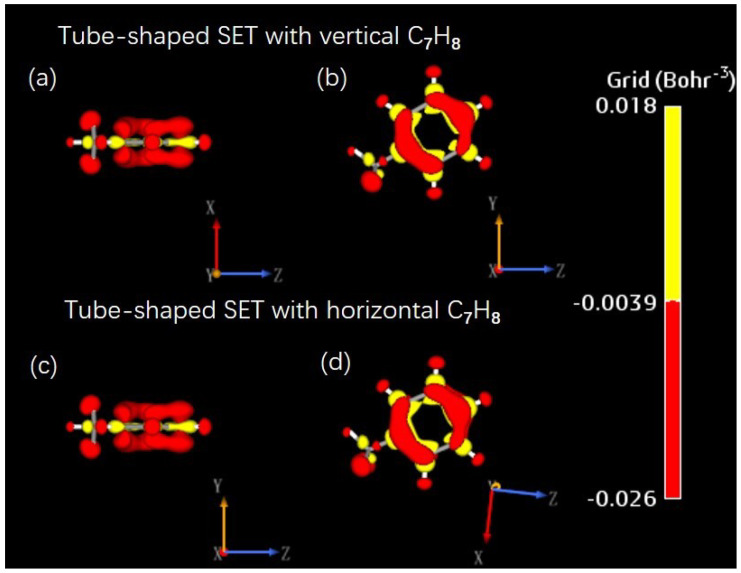
(**a**–**d**) Electron density of an additional net charge of +1 distributed on C${}_{7}$H${}_{8}$ under the tube-shaped SET environment. The upper and lower panels represent those with vertical and horizontal C${}_{7}$H${}_{8}$, respectively. The charge clouds are illustrated from different viewpoints in the left and right columns.

**Figure A7:**
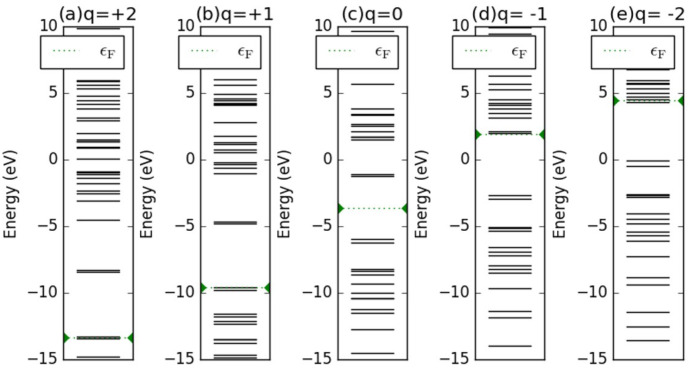
(**a**–**e**) Molecular energy spectra of horizontally oriented C${}_{7}$H${}_{8}$ with various charge states under the tube-shaped SET environment. The fermion energy level is marked in each subplot with green dots.

**Figure A8:**
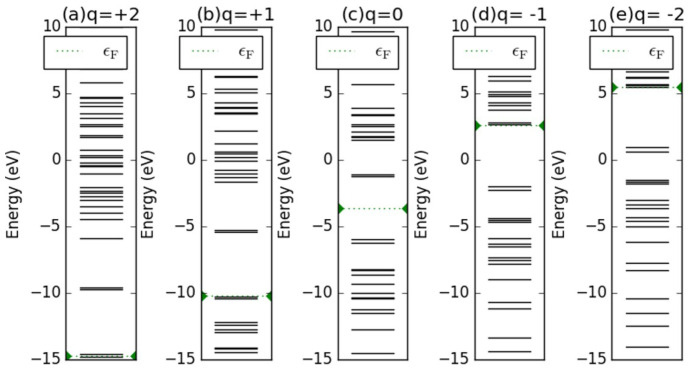
(**a**–**e**) Molecular energy spectra of vertically oriented C${}_{7}$H${}_{8}$ with various charge states under the conventional SET environment. The fermion energy level is marked in each subplot with green dots.

**Figure A9:**
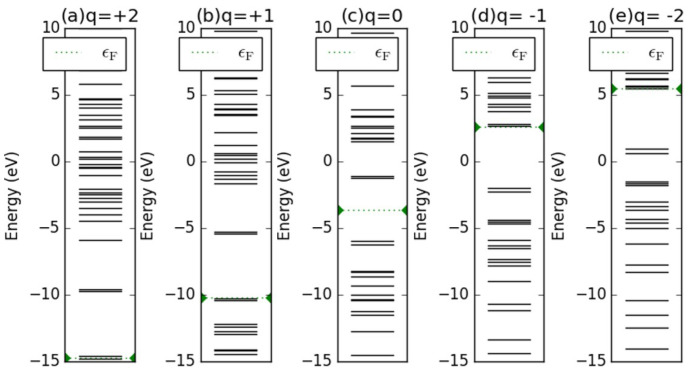
(**a**–**e**) Molecular energy spectra of horizontally oriented C${}_{7}$H${}_{8}$ with various charge states under the conventional SET environment. The fermion energy level is marked in each subplot with green dots.

In this section, we investigate the commonly met criteria pollutants molecules CO, NO${}_{2}$, O${}_{3}$, and SO${}_{2}$[46], as well as several typical hazardous pollutants molecules CH${}_{2}$O, C${}_{6}$H${}_{6}$, C${}_{7}$H${}_{8}$, C${}_{8}$H${}_{10}$, and C${}_{10}$H${}_{8}$, as examples of polycyclic aromatic hydrocarbons [47]. The molecules and the SET configurations are shown in Appendix B. The total energy of the investigated criteria pollutant and hazardous air pollutant molecules in the electrostatic environment is shown in Figure A4 and Figure A5 of Appendix C, respectively. Based on these total energies, the charge stability diagrams for the SET configurations with the criteria and hazardous air pollutant molecules were obtained and are illustrated in Figure 6 and Figure 7, respectively. The individual diagrams of the studied molecules had specific patterns, and the corresponding criteria pollutant and hazardous air pollutant molecules could be identified easily after the measured signals were analyzed by the data acquisition subsystem in Figure 1. The transport performance in the SET device was primarily decided by the electronic structure of the island molecule. The electronic structures of each criteria pollutant or hazardous air pollutant molecule differed, as shown in Appendix D. The electronic levels of the island molecule were well maintained during the operation of the SET due to the weak coupling between the electrodes and the molecule, and the pattern of the charge stability diagram was determined by the molecular electronics. Thus, the diagram can represent the intrinsic and unique features for each criteria pollutant and hazardous air pollutant molecule and can be treated as fingerprints of the molecules. For example, the size of the central Coulomb diamond in the charge stability diagram varied for all the studied molecules, as shown in Table A1 in Appendix B.

In addition, we investigated the coupling between the aforementioned molecules and the gate in the proposed device, and the results are shown in Table 2. The linear gate–island coupling dominated the interaction between the gate and air pollutant molecules, indicating that contribution of electrical polarization in the tube-shaped SET was negligible. The coupling strengths for each investigated molecule differed, further confirming the validity of the tube-shaped SET for identification of air pollutant molecules.

### 3.3. Quantification of Air Pollutants

Identification and quantification of air pollutants are the two major goals of testing a sample of ambient air. Traditionally, the entire process includes multiple stages, and different pieces of equipment are used in identification and quantification stages. For instance, the early procedures for the detection and quantitative measurements of the amount of SO${}_{2}$ varied, and different measurement devices were used in each process [9].

In our case, the proposed setup for the identification can be utilized for quantification as well. As shown in Figure 1, the quantification can be realized after the pollutant molecules go through the flow control component and flow to the center of the nanopore of the SET sensor. Using the air pollutant molecule as the island, the charge stability diagram can be produced after the operation of the SET device [21,22]. In the data acquisition subsystem, we can analyze the information gathered and obtain the concentration $c_{X}$ of each type of air pollutant *X*, as follows:

(2) $$\begin{array}{c} c_{X}=N_{X}m_{X}/V \end{array}$$

where $N_{X}$ denotes the number of molecules *X* identified during the measurement process, $m_{X}$ is the molecular weight of the molecule *X*, and *V* is the volume of air that went through the SET sensor for quantification. Each type of molecule must be well mixed in the tested sample air, and the testing time must be sufficiently long to obtain convergent results. To speed up the measurement process *M* times, the SET sensor can be replaced by an integrated sensor with *M* parallel SET devices. More details of the parallelization of SET can be found in [31].

Finally, the device can operate at room temperature. Normally, the characteristic charging energy will be larger than the thermal energy of the charge carriers only if the temperature is low enough, making the Coulomb blockade observable [48]. The charging energy $\Delta E^{i}\left(I\right)$ is needed to charge the island with one elementary charge. As listed in Table A2 of Appendix C, the energies used to charge different air pollutant molecules are sufficiently high ($k_{b}T_{300K}\ll \left|\Delta E^{i}\left(I\right)\right|$) to avoid the electron transport induced by the thermal fluctuations, in agreement with other molecule SET papers [30,31]. The SET device can thus operate over a large temperature range, including room temperature.

## 4. Conclusions

In the present work, based on the newly designed tube-shaped SET, an air pollutant measurement system was proposed to examine the air pollutants at the molecular level. First, we compared our new SET sensor with the conventional one. In each SET, the molecule was treated as the island after flowing to the center of the SET nanopore. The island molecule was placed along the *z*-axis with different orientations, i.e., either vertically or horizontally to the *xy*-plain in the studied cases. Operation and performance analysis of the SET sensor was performed using DFT-based ab initio calculations. We then studied the electronic properties of the island molecule under each SET environment, including the charge stability diagrams, electron densities, molecular energy spectra, total energies as functions of the gate voltages, and gate–island coupling. The effect of molecular orientation on the performance of the tube-shaped SET was found to be negligible, while it was not negligible for the conventional SET, demonstrating the advantage of our new sensor. Second, the new device was used to identify commonly encountered air pollutant molecules. The calculated charge stability diagrams were unique for each molecule, and thus they can be used as a fingerprint for detection. Third, we showed that the device prototype could identify and quantify the criteria pollutants and hazardous air pollutants in a unified framework. Our device was illustrated to operate over a broad range around room temperature, owing to the high charging energy of the molecules within it. In conclusion, this tube-shaped SET sensor has many potential applications. Its great versatility, high sensitivity, and elevated temperature of operation are significant advantages, making this SET-based detector a promising candidate for testing air quality at the molecular level. Research on the applications of the proposed device for testing other toxic molecules and designing more flexible detection setups for the dynamic sensing of air pollutant molecules is ongoing.

## Figures and Tables

**Table 1:** Gate coupling strengths between gates and island molecules.

SET Configuration			Gate-Island Coupling Strength	
SET Structure	ISLAND Molecule	Molecule Posture	Linear *α*	Quadratic *β* (eV^−1^)
Conventional	C${}_{7}$H${}_{8}$	Vertical	0.3417	−0.0056
Conventional	C${}_{7}$H${}_{8}$	Horizontal	0.3004	−0.0023
Tube−shaped	C${}_{7}$H${}_{8}$	Vertical	0.7962	−0.0006
Tube−shaped	C${}_{7}$H${}_{8}$	Horizontal	0.7962	−0.0006

**Table 2:** Gate coupling strengths between gates and island molecules of tube-shaped SET configurations.

Island Molecule	Gate–Island Coupling Strength	
	Linear α	Quadratic *β* (eV^−1^)
CO	0.8020	0.0000
NO${}_{2}$	0.8029	0.0000
O${}_{3}$	0.8138	0.0000
SO${}_{2}$	0.8005	−0.0001
CH${}_{2}$O	0.8035	0.0000
C${}_{6}$H${}_{6}$	0.8031	−0.0004
C${}_{8}$H${}_{10}$	0.7954	−0.0009
C${}_{10}$H${}_{8}$	0.7768	−0.0009

**Figure A1:**
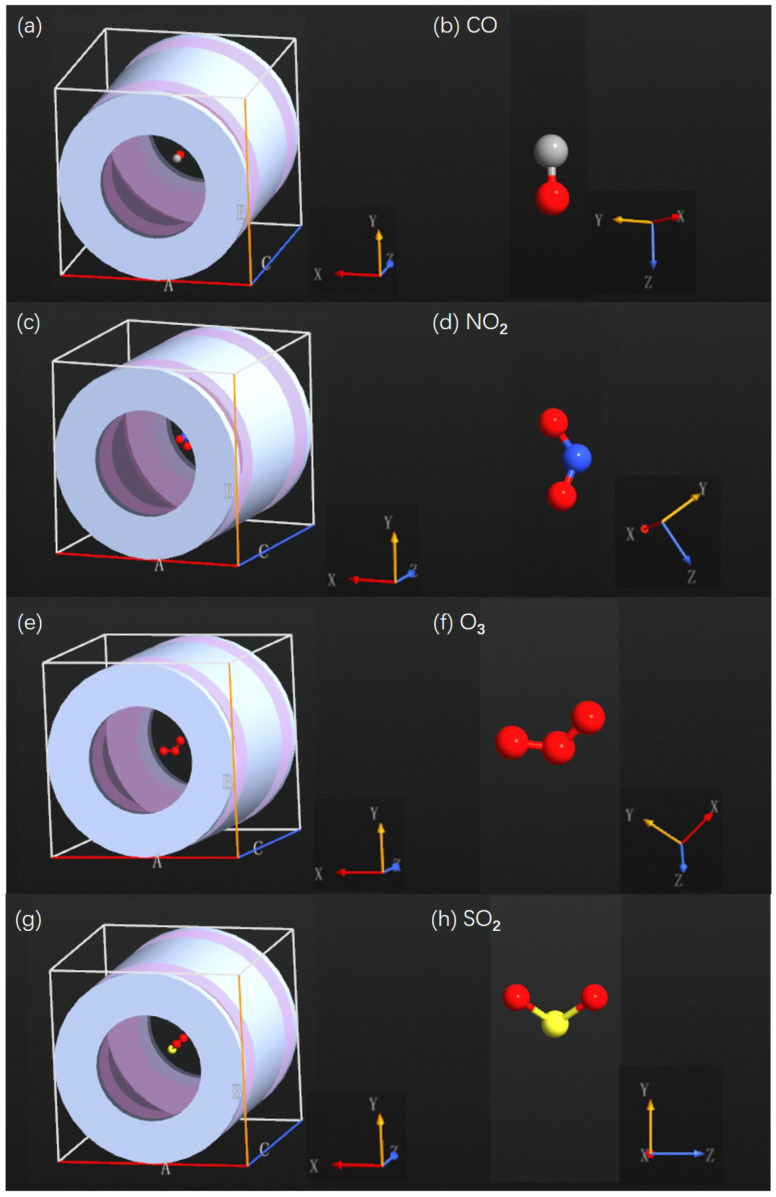
(**a**) Tube-shaped SET with a CO molecule as the island, (**b**) CO molecule, (**c**) tube-shaped SET with an NO${}_{2}$ molecule as the island, (**d**) NO${}_{2}$ molecule, (**e**) tube-shaped SET with an O${}_{3}$ molecule as the island, (**f**) O${}_{3}$ molecule, (**g**) tube-shaped ET with an SO${}_{2}$ molecule as the island, and (**h**) SO${}_{2}$ molecule.

**Figure A2:**
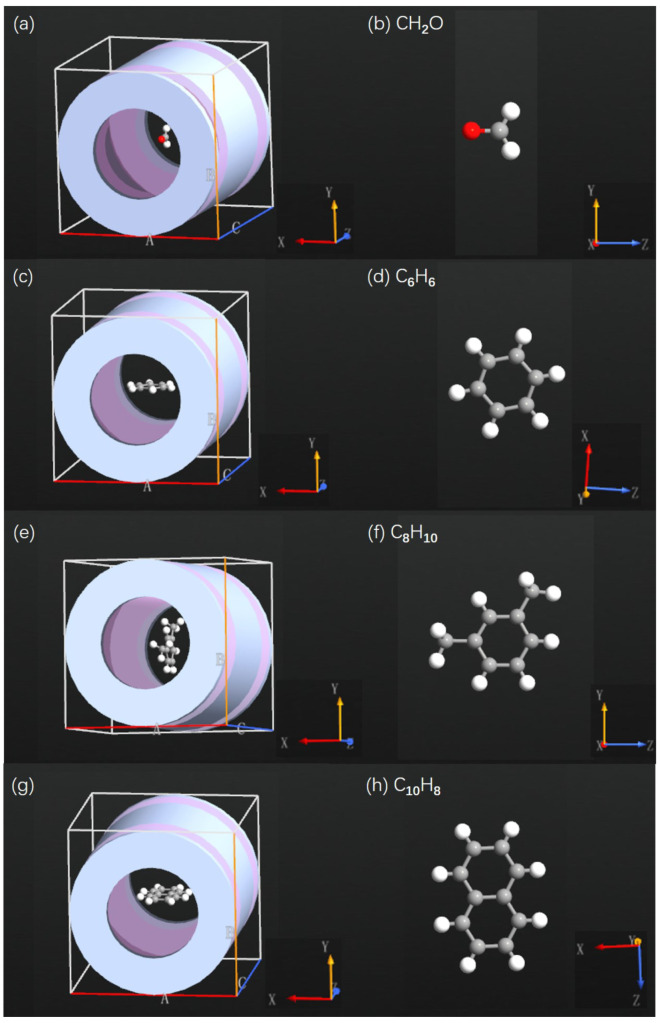
(**a**) Tube-shaped SET with a CH${}_{2}$O molecule as the island, (**b**) CH${}_{2}$O molecule, (**c**) tube-shaped SET with a C${}_{6}$H${}_{6}$ molecule as the island, (**d**) C${}_{6}$H${}_{6}$ molecule, (**e**) tube-shaped SET with a C${}_{8}$H${}_{10}$ molecule as the island, (**f**) C${}_{8}$H${}_{10}$ molecule, (**g**) tube-shaped SET with a C${}_{10}$H${}_{8}$ molecule as the island, and (**h**) C${}_{10}$H${}_{8}$ molecule.

**Figure A10:**
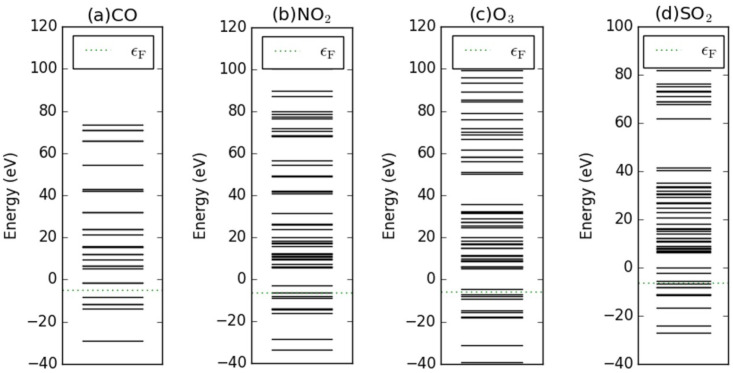
Molecular energy spectra of neutralized criteria pollutant molecules of (**a**) CO, (**b**) NO${}_{2}$, (**c**) O${}_{3}$, and (**d**) SO${}_{2}$ in tube-shaped SET environment. The fermion energy level is marked in each subplot with green dots.

**Figure A11:**
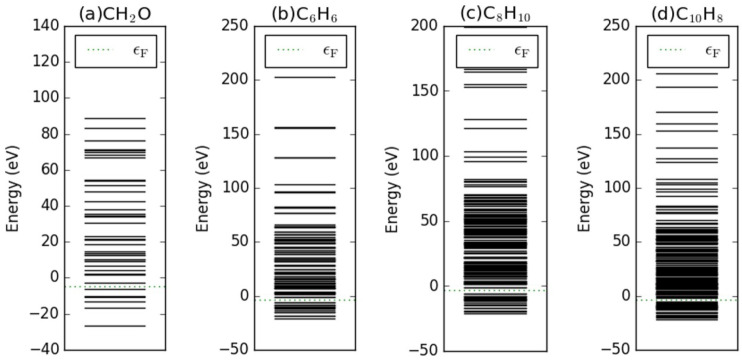
Molecular energy spectra of neutralized hazardous air pollutant molecules of (**a**) CH${}_{2}$O, (**b**) C${}_{6}$H${}_{6}$, (**c**) C${}_{8}$H${}_{10}$, and (**d**) C${}_{10}$H${}_{8}$ in tube-shaped SET environment. The fermion energy level is marked in each subplot with green dots.

## Data Availability

The datasets used and/or analyzed during the current study are available from the corresponding author on reasonable request.

## Appendix A. Working Principle of SET for Detection of Air Pollutant Molecules

Coupling between the source/drain electrodes and the island was weak within the SET device. An electron would tunnel from the source to the drain electrode via the island. During the transport, because of the localization and long stay of an electron in the island, the original and final quantum states of the electron would become uncorrelated, and the electron would undergo subsequent tunneling instead of coherent tunneling. This process is known as the Coulomb blockade.

In detail, when the electron tunnels through the barrier between the source electrode and the island, the energy of the electron on the island must be lower than that of the electron in the source electrode,

(A1) $$E^{s}\left(S\right)+E^{i}\left(I\right)\geq E^{s}\left(S-1\right)+E^{i}\left(I+1\right),$$

where *S* and *I* are the initial numbers of electrons on the island and in the source electrode, respectively. $E^{s}\left(q\right)$, $E^{i}\left(q\right)$, and $E^{d}\left(q\right)$ give the total energy of the source electrode, island, and drain electrode as functions of the number of electrons *q*, respectively.

Similarly, if the electron transfers from the island to the drain electrode, the energy of the electron on the island must be higher than that in the drain electrode. Moreover, to move the electron from the island to the drain electrode, it must have a lower energy in the drain electrode,

(A2) $$E^{d}\left(D\right)+E^{i}\left(I+1\right)\geq E^{d}\left(D+1\right)+E^{i}\left(I\right),$$

where *D* is the initial number of electrons in the drain electrode.

The maximum energy of the electron in the source electrode is $-W+eV_{b}/2$, where *W* is the work function of the electrode and $V_{b}$ is the applied bias. Assuming that the electron with maximum energy tunnels onto the island, then the energy is

(A3) $$E^{s}\left(S\right)-E^{s}\left(S-1\right)=-W+eV_{b}/2.$$

Using the above tunneling criterion, we obtain the condition

(A4) $$-W+eV_{b}/2+E^{i\;}\left(I\right)\geq E^{i}\left(I+1\right).$$

Similarly, $-W-eV_{b}/2$ is the minimum energy of an electron in the drain electrode, and thus

(A5) $$E^{i}\left(I+1\right)\geq -W-eV_{b}/2+E^{i}\left(I\right).$$

The requirement for a current to flow in the device is therefore

(A6) $$e|V_{b}|/2\geq \Delta E^{i}\left(I\right)+W\geq -e\left|V_{b}\right|/2,$$

where $\Delta E^{i}\left(I\right)=E^{i}\left(I+1\right)-E^{i}\left(I\right)$ is the charging energy of the island.

In this work, the charging energies of different molecular SETs were calculated, and Equation (A6) was used to obtain the so-called charge stability diagram, which shows the number of charge states inside the bias window as a function of the gate and source–drain voltages. In detail, the sizes of the central diamonds in the charge stability diagrams for the investigated air pollutant molecules are shown in Table A1.

**Table A1:** Size of the central diamond in the charge stability diagram of tube-shaped SET configurations with an individual island molecule.

Island Molecule	CO	NO${}_{2}$	O${}_{3}$	SO${}_{2}$	CH${}_{2}$O	C${}_{6}$H${}_{6}$	C${}_{7}$H${}_{8}$	C${}_{8}$H${}_{10}$	C${}_{10}$H${}_{8}$
Length of a diagonal line in the central diamond along the gate voltage axis (V)	18.5	9.9	12.3	9.3	13.1	10.4	9.9	9.4	7.4
Length of a diagonal line in the central diamond along the source-drain bias (V)	29.6	16.0	20.0	14.8	21	16.8	15.6	14.9	11.2

## Appendix B. SET Configurations with Various Air Pollutant Molecules

The SET configurations for investigating criteria pollutant molecules and hazardous air pollutant molecules are illustrated in Figure A1 and Figure A2, respectively.

## Appendix C. Total Energy as Function of Gate Voltage

The total energy of C${}_{7}$H${}_{8}$ in each SET environment as a function of the gate voltage is shown in Figure A3. The total energies of the criteria pollutant molecules and hazardous air pollutant molecules in the tube-shaped SET environment as functions of the gate voltage are displayed in Figure A4 and Figure A5, respectively.

The charging energies of the island molecules were deduced from the total energies obtained from the first-principle calculations, as illustrated in Table A2.

**Table A2:** Charging energy for state *I* is $\Delta E^{i}\left(I\right)=E^{i}\left(I+1\right)-E^{i}\left(I\right)$ at zero gate voltage, where $E^{i}\left(I\right)$ is the total energy of the island molecule with a net charge *I*. The charging energy of the investigated air pollutant molecule for various states are shown.

Island Molecule	CO	NO${}_{2}$	O${}_{3}$	SO${}_{2}$	CH${}_{2}$O	C${}_{6}$H${}_{6}$	C${}_{7}$H${}_{8}$	C${}_{8}$H${}_{10}$	C${}_{10}$H${}_{8}$
$\Delta E^{i}\left(+1\right)$ (eV)	23.7019	20.3182	19.9398	17.6599	19.4065	12.3559	11.4820	10.8229	10.0120
$\Delta E^{i}\left(0\right)$ (eV)	13.2354	10.4107	10.8392	10.1616	10.2545	8.1345	7.6662	7.3421	6.7887
$\Delta E^{i}\left(-1\right)$ (eV)	−1.5923	2.4329	0.8093	2.6771	−0.2429	−0.2895	−0.2405	−0.1665	1.0380
$\Delta E^{i}\left(-2\right)$ (eV)	−7.4722	−6.8920	−6.2228	−3.0606	−6.1735	−3.8604	−3.2489	−2.8812	−1.9152

## Appendix D. Electronic Structures of the Investigated Molecules

The molecular energy spectra of the investigated criteria pollutant and hazardous pollutant molecules are illustrated in Figure A10 and Figure A11, respectively. It is evident that these spectra differed, indicating the validity of the tube-shaped SET for identifying corresponding molecules.
